# Multi-Detector Computed Tomography Imaging Techniques in Arterial Injuries

**DOI:** 10.3390/jcm7050088

**Published:** 2018-04-24

**Authors:** Cameron Adler, Patrick T. Hangge, Hassan Albadawi, M-Grace Knuttinen, Sadeer J. Alzubaidi, Sailendra G. Naidu, Rahmi Oklu

**Affiliations:** 1 Department of Vascular and Interventional Radiology, Minimally Invasive Therapeutics Laboratory, Mayo Clinic, Phoenix, AZ 85054, USA; adler.cameron@mayo.edu (C.A.); Hangge.Patrick@mayo.edu (P.T.H.); Albadawi.Hassan@mayo.edu (H.A.); knuttinen.grace@mayo.edu (M.-G.K.); Alzubaidi.Sadeer@mayo.edu (S.J.A.); naidu.sailen@mayo.edu (S.G.N.); 2 Department of General Surgery, Mayo Clinic, Phoenix, AZ 85054, USA

**Keywords:** computed tomography, CT, angiography, arterial injury, radiology, cross-sectional, imaging

## Abstract

Cross-sectional imaging has become a critical aspect in the evaluation of arterial injuries. In particular, angiography using computed tomography (CT) is the imaging of choice. A variety of techniques and options are available when evaluating for arterial injuries. Techniques involve contrast bolus, various phases of contrast enhancement, multiplanar reconstruction, volume rendering, and maximum intensity projection. After the images are rendered, a variety of features may be seen that diagnose the injury. This article provides a general overview of the techniques, important findings, and pitfalls in cross sectional imaging of arterial imaging, particularly in relation to computed tomography. In addition, the future directions of computed tomography, including a few techniques in the process of development, is also discussed.

## 1. Introduction

Arterial injuries are seen in a variety of settings, including blunt trauma, penetrating trauma, complications of percutaneous access, and surgical complications. If not diagnosed quickly, these injuries can be devastating, resulting in hemorrhage, hemorrhagic shock, and even death [1]. As cross-sectional imaging techniques have improved, their use has become widespread and they have become primary tools in the diagnosis of arterial injuries, especially prior to treatment. Rapid identification of both the location and severity of an injury are crucial for institution of rapid treatment and prevention of morbidity and mortality due to arterial injury [2].

With regard to cross-sectional imaging in the setting of arterial injury, computed tomography (CT) is typically the cross-sectional imaging technique of choice [3,4]. Here, we will discuss the basic principles of the CT imaging technique in arterial injury. Parameters of imaging protocols are dependent upon the area of the body being examined and available equipment, leading to extensive variability among protocols. As such, we will focus on the important principles and general tools available in arterial imaging, rather than detailing specific protocols for all regions of the body, which is beyond the scope of this article. In addition, the clinical indications for imaging evaluation of an arterial injury are innumerable and include trauma, iatrogenic injury, a wide range of symptoms, specific comorbidities, and more. Since we aim to provide a general overview of arterial imaging techniques and findings in the setting of injury, we will not discuss clinical indications in detail. 

## 2. Technique

### 2.1. Contrast Bolus

In assessing arterial injury, the timing of imaging and the use of contrast are important factors. When injecting iodinated contrast, an injection rate of 4–5 mL per second is standard [2]. The total volume of contrast is variable and depends on the area of the body being examined, along with the size of the patient, with the total volume of non-ionic iodinated contrast typically between 100 and 150 mL of 300–350 mgI/mL [4,5]. Image acquisition timing is usually determined using fixed time delay, test bolus, or bolus tracking. Multiple factors need to be considered when determining bolus and acquisition timing, including time to peak enhancement, location of target vessels, injection site, saline bolus, cardiac output, imaging duration, coverage of imaging, and patient-related limitations. All of these factors can alter bolus hemodynamics and alter the optimal imaging time following bolus administration [6].

The fixed time-delay technique acquires arterial phase images after a fixed amount of time passes following the administration of the contrast bolus. It is the simplest of the three acquisition timing methods for arteriography. The time delay following the bolus injection is constant and specific to the area of the body being examined. This method is adequate for the majority of the population, but is problematic when there is abnormal anatomy, abnormal cardiac function, or severe arterial disease, all of which can significantly alter the transit time of blood. Despite known limitations, there is typically no significant difference in arterial phase enhancement with the fixed time-delay imaging technique in comparison to bolus tracking [7].

A test bolus involves the injection of a small bolus of contrast, typically 15–20 mL of an iodinated contrast agent, and observation of the major artery with a region of interest. The major artery is monitored to determine when the peak attenuation is obtained in that artery. The time from administration of contrast to peak attenuation in the artery of interest is recorded and used as the scan delay time following administration of the full bolus for the diagnostic exam. Subsequently, the full bolus of contrast is administered and image acquisition begins after the time delay determined from the test bolus has elapsed. Once image acquisition is initiated, the entire acquisition occurs at a constant rate. Prior studies have shown that peak enhancement from the timing test bolus is similar to the peak enhancement timing of the full bolus [8,9]. The test bolus method is rarely used today, as it requires the administration of a larger total volume of contrast.

**Figure 1:**
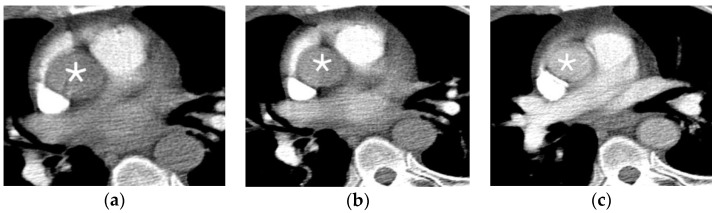
Consecutive axial images acquired during bolus tracking. The region of interest was placed over the ascending aorta, as marked by an asterisk. (**a**) Ascending aorta with attenuation greater than 50 Hounsfield units; (**b**) Ascending aorta with attenuation greater than 50 and less than 100 Hounsfield units; (**c**) Ascending aorta with attenuation greater than 120 Hounsfield units, at which point image acquisition began.

The technique for bolus tracking is similar to a test bolus, however without the need for the initial smaller bolus. Prior to the injection of the bolus, the region of interest is placed over the major artery in the region of the body being examined and the attenuation is monitored. Once the selected attenuation threshold is reached, image acquisition is triggered immediately or following an additional short delay (Figure 1). Bolus tracking is commonly used today. Although bolus tracking is generally preferred, the fixed time delay used with this method varies based on the scan time, scanning speed, and injection duration [10]. Bolus tracking tends to be preferred in patients who are expected to have significantly abnormal circulation times, as circulation time is a crucial component of image acquisition timing [6]. In patients with significantly abnormal circulation times, bolus tracking prevents image acquisition from being initiated prior to the bolus reaching the area of interest.

Following contrast administration, a small saline bolus should be administered. The saline bolus flushes the contrast through the catheter, increasing the utility of the contrast, and dilutes the contrast bolus in the veins directly after injection. Diluting the portion of the bolus remaining in the veins at the time of imaging reduces the beam-hardening artifacts that can result from the high concentration of contrast in these veins after administration of contrast [2].

### 2.2. Imaging Phases

Multiple phases of computed tomography imaging can be used to assess for arterial injury: non-contrast, arterial, venous, and delayed. Each of these phases has unique utility, but also has its own limitations. Understanding the utility and limitations of each phase is paramount to providing a thorough evaluation and accurate diagnosis for the patient in question. Non-contrast-enhanced imaging can be of great utility in the setting of suspected intramural hematoma of the aorta. In addition, it can be helpful in certain post-processing techniques. Arterial phase imaging is the primary component of arterial imaging and provides excellent assessment of the arterial lumen and may demonstrate a variety of arterial injuries, as described in Section 2.4. Venous or delayed phase imaging may provide additional information in patients with arterial injury of smaller vessels or slower, low-volume hemorrhage. In these situations, the contrast-enhanced blood can be seen accumulating over time in the case of extravasation and may be helpful for differentiating extravasation from a pseudoaneurysm in certain cases [5].

### 2.3. Image Acquisition and Reconstruction

Multidetector helical CT is the technology of choice for image acquisition in arterial cross-sectional imaging. Mutidetector CT allows large volumes to be covered more rapidly than prior iterations of CT, including fan-beam and single-detector row CT. More detector rows allow for a reduced time of image acquisition and prevents contrast enhancement of the venous system before the entire scan is acquired [2].

When acquiring images, electrocardiographic (ECG) gating may be used for thoracic arterial imaging to prevent motion artifacts related to cardiac motion, although is not required when using a multi-detector CT of 64 rows or greater. ECG gating times image acquisition with respect to the patient’s ECG so that images can be reconstructed at a single point in the cardiac cycle. ECG gating is an important mechanism for reducing motion artifacts, especially when imaging suspected aortic injuries [11].

**Figure 2:**
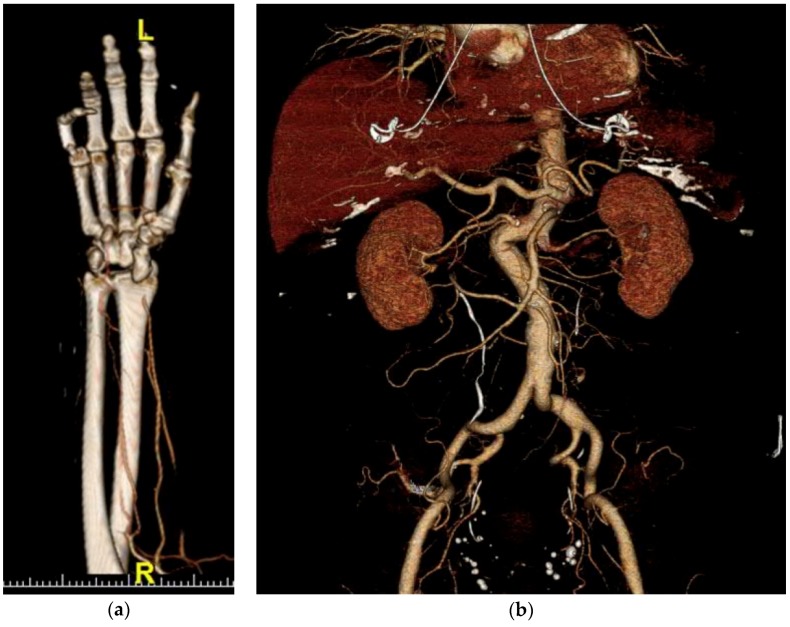
Volume rendering examples: (**a**) Volume rendering of distal radial artery occlusion; (**b**) Volume rendering of abdominal arteries.

There are a few image reconstruction and post-processing techniques commonly used in arterial imaging. In image reconstruction, multiplanar reformatting (MPR) is always performed. MPR provides a different plane of view when scrolling through the images. Typically, MPR will include axial, sagittal, and coronal reconstructions. In the case of aortic injury, sagittal oblique reconstructions will also be obtained in order to see a longitudinal view of the thoracic aorta that includes the ascending aorta, aortic arch, and descending aorta in individual images. Post-processing techniques often involve maximum intensity projection (MIP), volume rendering, and bone subtraction angiography. Of those post-processing techniques, MIP is the most common. MIP takes multiple thin slices of images, combines them, and uses only the highest-attenuation data voxel among the overlapping voxels. MIP excels at enhancing the visualization of high-intensity structures, like contrast-enhanced arteries. One of the biggest advantages of MIP reconstructions is the ability to enhance the visualization of small branch arteries. Volume rendering uses complex post-processing algorithms to create a three-dimensional representation of the structures that accurately maintains the special relationships of the structures in three dimensions and can be manipulated in three dimensions, as seen in Figure 2. Bone subtraction angiography uses a non-contrast image set, identifies the bone, and subtracts it from the contrast-enhanced image set. This subtraction leaves only soft tissues and highly attenuated arteries in the remaining image set. This provides a detailed and unobstructed evaluation of contrast-enhanced arteries, given that the only other remaining structures are of very low attenuation [2,12].

### 2.4. Imaging Patterns in Arterial Injury

Regardless of the mechanism or location of the arterial injury, there is a group of very important injury patterns to search for in the setting of suspected arterial injury. Despite the fact that many clinicians will ask specifically about extravasation, other findings of arterial injury are common. These other findings include abrupt arterial narrowing, occlusion, dissection, intramural hematoma, pseudoaneurysm, and arteriovenous fistula.

#### 2.4.1. Active Arterial Extravasation

**Figure 3:**
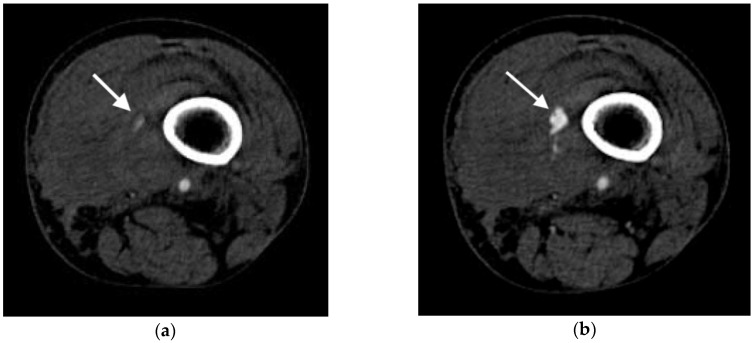
Axial images of active extravasation of contrast into a medial left thigh hematoma following traumatic injury to the left leg: (**a**) Small volume of contrast extravasation seen during the arterial phase (arrow); (**b**) Increase in size of the collection of extravasated contrast in the delayed phase imaging (arrow).

Active arterial extravasation is usually the first sign that a radiologist is asked about in the setting of suspected arterial injury. Extravasation is visualized as a region of hyperattenuation outside of the injured artery, typically without a well-defined border in later phases of contrast-enhanced imaging. The level of attenuation in the pooling contrast-enhanced blood should be similar to or greater than the aorta when viewed in arterial phases. In the portal venous, venous, and delayed phases, the region of increased attenuation should increase in size as the image sequences become more delayed and the degree of attenuation should be greater than that of the aorta in these phases. Figure 3 demonstrates active extravasation in the arterial and delayed phases. It should be noted that venous injury may also lead to extravasation of contrast and have some overlap of features with arterial injury. Venous extravasation may be differentiated from arterial extravasation by the lack of extravascular pooling of contrast in the arterial phase images and by a lesser degree of attenuation of the region of pooling relative to arterial extravasation. In order for the extravasation to be visible, the attenuation of the contrast-enhanced blood needs to be higher than the surrounding blood and hematoma. Prior studies have demonstrated that active extravasation has an attenuation range from 85 to 370 Hounsfield units, while clotted blood has a lower attenuation of 40–70 Hounsfield units [3]. As the extravasation occurs, the contrast-enhanced blood will mix with the surrounding blood and the degree of attenuation will gradually decrease. This results in the highest area of extravascular attenuation being the area closest to the actual location of injury, although this effect is not always visible in CT imaging [5,13].

#### 2.4.2. Arterial Occlusion or Stenosis

**Figure 4:**
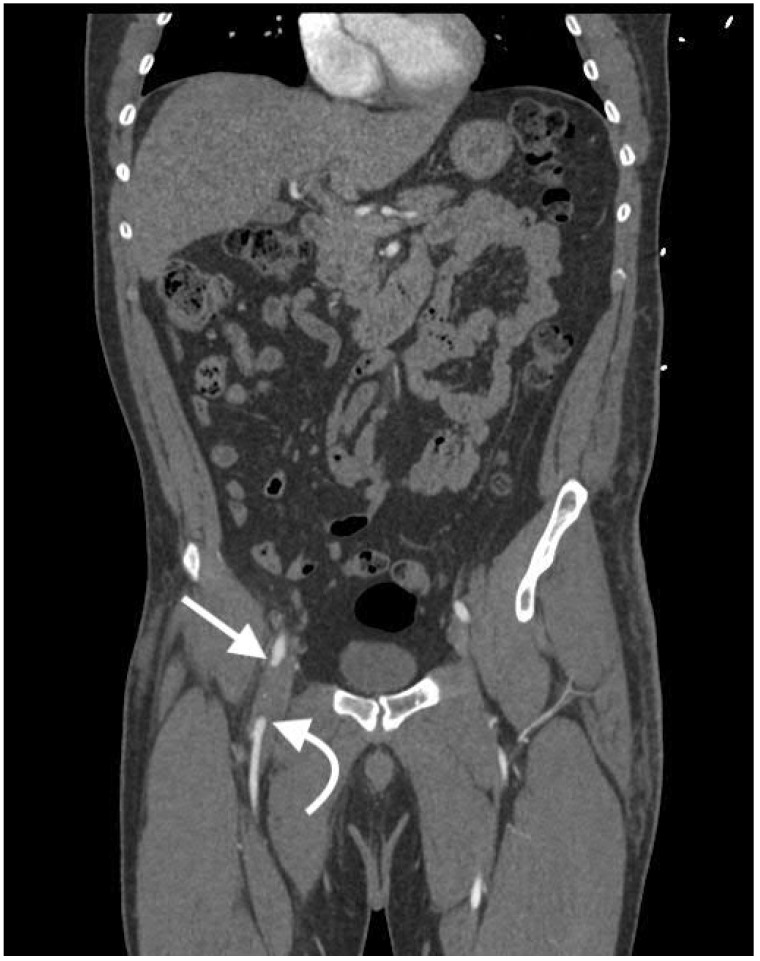
Coronal image of right common femoral artery occlusion (arrow) with distal reconstitution (curved arrow) following traumatic injury to the pelvis.

**Figure 5:**
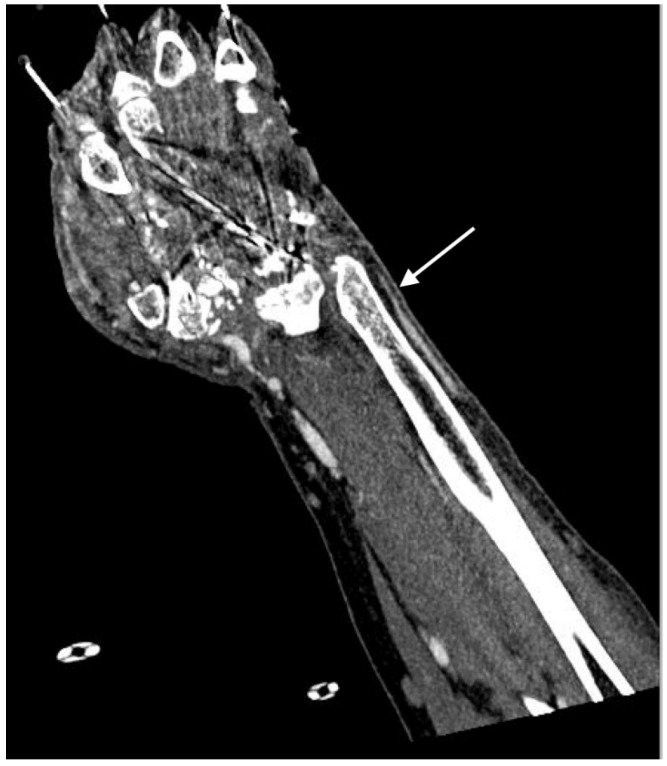
Coronal image of right distal ulnar artery stenosis following traumatic injury to the wrist and hand.

Arterial occlusion and stenosis have a wide array of causes, including intramural hematoma, vasospasm, intimal injuries, thrombus, embolus, and mass effect. Despite the varying causes, occlusion and stenosis share a somewhat similar appearance. Arterial occlusion presents as an abrupt end of the vessel, as seen in Figure 4. In comparison, arterial stenosis presents as a gradual tapering or focal decrease in caliber of the artery, as seen in Figure 5. Although the occlusion may lead to a segment of unenhanced artery, the distal artery may still reconstitute and distal reconstitution should not be mistaken for lack of injury, as demonstrated by Figure 4. Furthermore, the tapering or narrowing does not need to be symmetrical, even if the specific cause of the narrowing typically presents circumferentially. In the setting of occlusion or stenosis due to mass effect, cross-sectional imaging has the added benefit of identifying the specific cause of the mass effect, such as hematoma, in addition to identifying the arterial injury. This added benefit leads to treatment of the primary problem and prevents unnecessary arterial procedures that fail to address the primary cause of the arterial injury [14,15].

#### 2.4.3. Arterial Dissection

**Figure 6:**
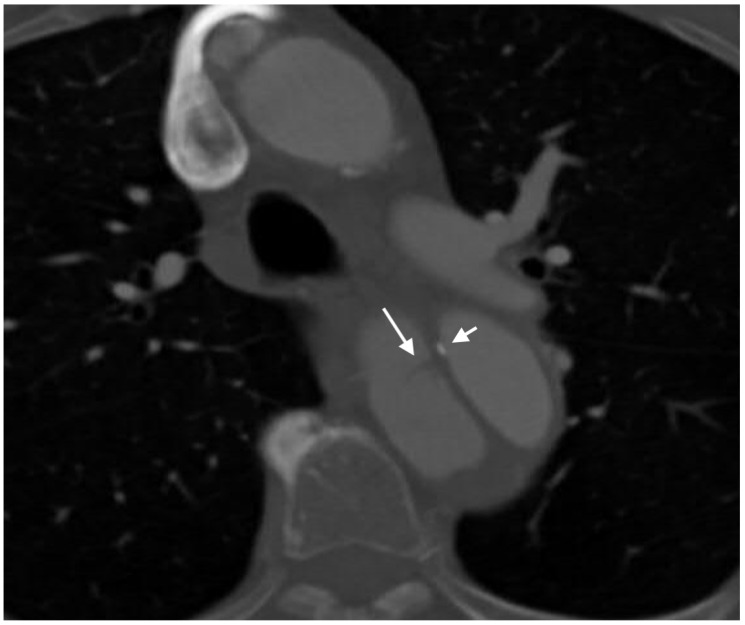
Axial image of type A aortic dissection demonstrating both the cobweb sign (long arrow) and eccentric calcification in the dissection flap (short arrow) in the descending aorta. Note that the eccentric calcification abuts the true lumen.

**Figure 7:**
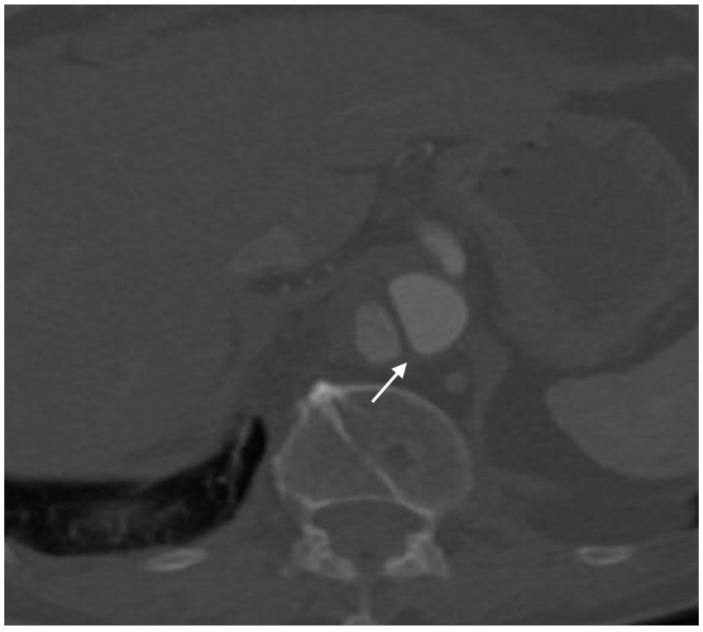
Axial image of aortic dissection demonstrating the beak sign (arrow). Note the acute angle between the dissection flap and the outer wall.

Arterial dissection classically presents as an intraluminal flap, typically a thin layer of intima and media seen traversing the lumen of the artery that leads to the identification of two lumens in the artery: a true and false lumen. This type of injury is typically only seen in larger vessels, with Miller-Thomas et al. reporting that they had never seen an arterial intimal flap while imaging the extremities in the setting of suspected arterial injury [14]. While the intraluminal flap may have a classic appearance, some cases are not so straightforward. For example, circumferential dissection can lead to a lumen within a lumen, giving a targetoid appearance in axial cross-section and an intussusception-like appearance in longitudinal cross-section of the vessel, often referred to as the “windsock” deformity. CT angiography can also identify the extent of the dissection and the branching vessels involved, and can be used to help identify the true and false lumens, all of which are important in treatment planning. Features that may help identify the false lumen include the “cobweb sign,” “beak sign,” eccentric wall calcification, and the size of both lumens at the midpoint of the dissection, all of which were described and evaluated by LePage et al. in the setting of aortic dissection. The cobweb sign appears as wispy strings of intimal tissue that dangle off of the walls of the false lumen, as in Figure 6. These wispy strings are thought to be strands of connective tissue from the arterial wall that are not completely fixed to the wall after the dissection occurs and are only seen within the false lumen. The beak sign, as seen in Figure 7, is an acute angle between the dissection flap and the outer wall and may be filled with contrast-enhanced blood or a hematoma with low attenuation. At the midpoint of the dissection, approximately 95% of aortic dissections will show a larger false lumen than true lumen. Eccentric calcification in the dissection flap, if seen, is usually noted on the true lumen side of the dissection flap, as demonstrated in Figure 6 [16].

#### 2.4.4. Intramural Hematoma

**Figure 8:**
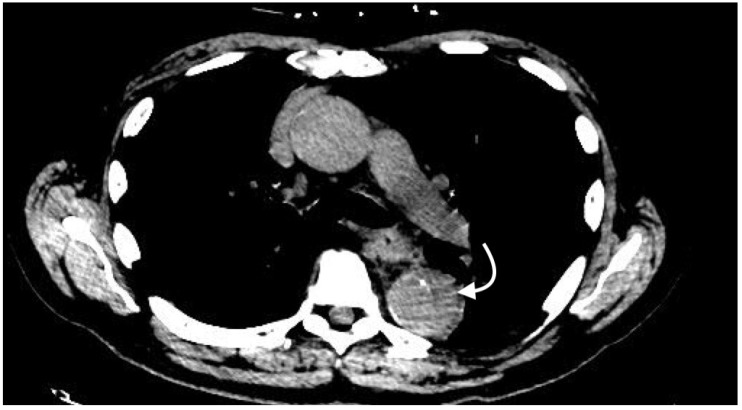
Axial non-contrast image demonstrating intramural hematoma (curved arrow) in the descending thoracic aorta. Note that the crescent-shaped intramural hematoma has higher attenuation than the thin portions of normal arterial wall surrounding it.

**Figure 9:**
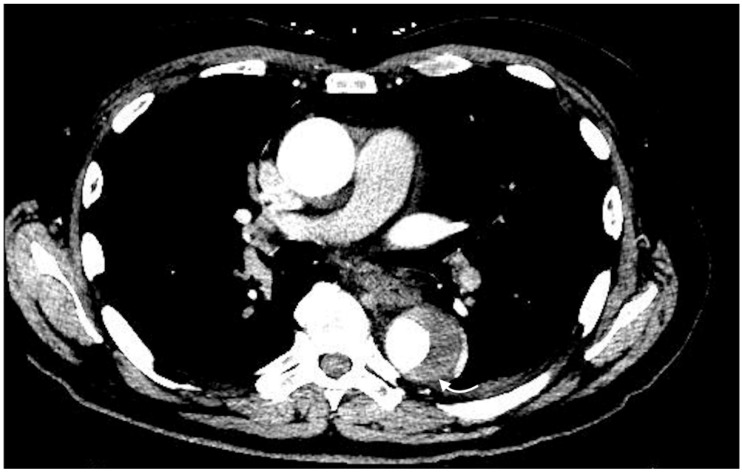
Axial contrast-enhanced arterial phase image demonstrating a crescent-shaped intramural hematoma (curved arrow) in the descending aorta.

Intramural hematoma can be a difficult injury to classify, especially when occurring within the aorta, as it is typically considered in the spectrum of injury known as acute aortic syndrome [17]. In smaller vessels, the intramural hematoma often cannot be differentiated from vasospasm, partial thrombosis, or small extramural hematoma [13]. Classically, intramural hematoma can be identified as a hyperattenuating focus with an either crescentic or circular shape that is present in the aortic wall in non-contrast-enhanced CT, as seen in Figure 8. In contrast-enhanced imaging, the intramural hematoma appears to be hypoattenuating relative to the lumen and may not appear different in attenuation from the remainder of the normal arterial wall, as seen in Figure 9 [18]. If calcifications are present, the intramural hematoma may be associated with focal displacement of intimal calcifications. Focal mural thickening from the intramural hematoma may also result in a decreased luminal diameter that will be visible in contrast-enhanced imaging. However, the hematoma itself will likely not be visible in the contrast-enhanced phases, even if the asymmetric thickening is prominent enough to result in luminal narrowing. This results in an appearance somewhat similar to the stenosis pattern described above. Visualization of this hyperattenuating focus may be impossible once contrast is given and may be the only sign of intramural hematoma. As discussed by Gutschow et al. intramural hematoma was formerly thought to be an entirely intramural process. However, continued improvement in imaging technology has allowed for identification of smaller tears in the intima on evaluation of some intramural hematomas, further blurring the definition of intramural hematoma in the setting of acute aortic syndrome. The small tears seen in newer CT scanners may explain the enhancement of some intramural hematomas seen in older contrast-enhanced imaging, suggesting that older technology did not have high enough resolution to identify the tear [17].

#### 2.4.5. Pseudoaneurysm

**Figure 10:**
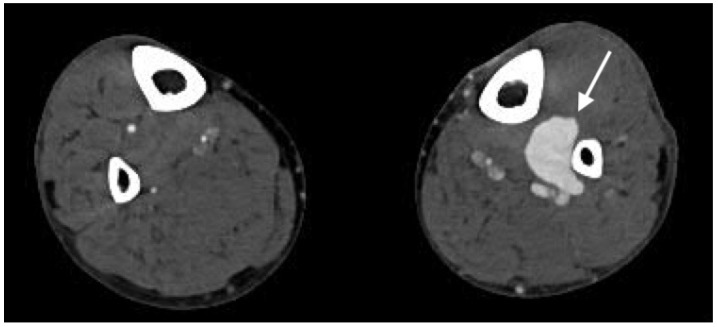
Axial image demonstrating large left peroneal artery pseudoaneurysm (arrow) following traumatic injury to the lower extremity.

A pseudoaneurysm appears as an outpouching of blood that communicates with the arterial lumen. It typically has a well-defined border that is either round, as seen in Figure 10, or lobulated [13]. The enhancement of the pseudoaneurysm generally follows that of arteries throughout all phases of imaging, a finding that is key to differentiating the pseudoaneurysm from extravasation. However, a portion of the pseudoaneurysm may fail to enhance, indicating thrombus contained within the pseudoaneurysm [19].

#### 2.4.6. Arteriovenous Fistula

**Figure 11:**
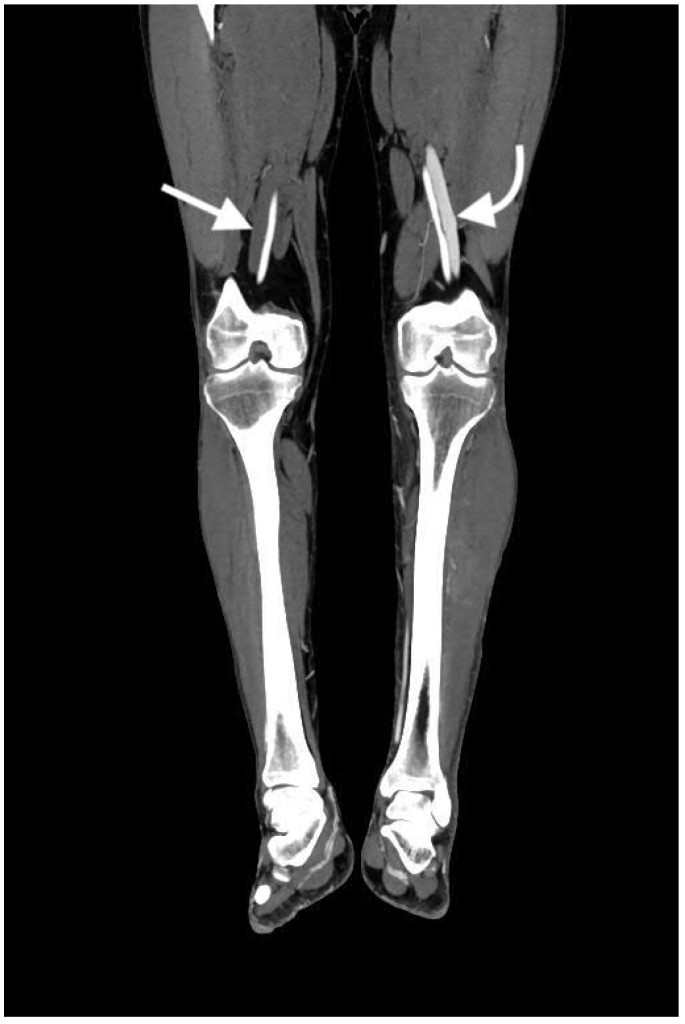
Coronal image of the bilateral lower extremities in the arterial phase following left calf trauma. There is opacification of the left femoral vein (curved arrow) in the arterial phase, indicative of arteriovenous fistula. Note that the right femoral vein (arrow) is not opacified.

An arteriovenous fistula can be very difficult to detect. The most important feature for identifying an arteriovenous fistula is early filling of venous structures in arterial phase imaging, as seen in Figure 11. Visualization of the fistulous tract is diagnostic of the arteriovenous fistula, however the tract is often not seen in contrast-enhanced CT [15]. Any contrast-enhancing venous structure in arterial phase images should raise suspicion for an arteriovenous fistula, as venous structures should not opacify with contrast in arterial phases. With regard to the veins of the pelvis and extremities, it can be very helpful if both sides can be examined for symmetric enhancement. Asymmetric enhancement in the arterial phase can make early venous enhancement much more apparent. In the case of an arteriovenous fistula in an artery supplying an organ, there may be decreased parenchymal enhancement of that organ, due to shunting of the blood away from the organ [20,21].

### 2.5. Imaging Performance

CT has excellent performance in identification of arterial injuries, regardless of the region of the body affected or the mechanism of injury. Prospective evaluation has found sensitivity and specificity for arterial injury on CT angiography of the extremities to be as high as 95% and 87%, respectively [4,13]. Studies evaluating injury of the abdomen and pelvis have demonstrated sensitivity and specificity for arterial injuries as high as 97% and 95%, respectively [5]. For example, Pereira et al., demonstrated a sensitivity of 90% and specificity of 98.6% in the setting of hemorrhage following pelvic trauma [22]. In the setting of proximal extremity injuries, Soto et al. found a sensitivity of up to 95% and a specificity of up to 98% [15]. Inaba et al. demonstrated a sensitivity of 100% and specificity of 100% for patients treated for lower extremity trauma at their level I trauma center [23]. Gavant et al. examined the performance of CT angiography in thoracic aortic rupture resulting from blunt trauma and found a sensitivity of 100% and specificity of 81.7% [24]. In the setting of penetrating neck trauma, Munera et al., demonstrated a mean sensitivity of 90% and specificity of 100% [1]. Overall, regardless of the region of the body being examined and the mechanism of injury, CT angiography demonstrates excellent sensitivity and specificity in detection of arterial injuries.

### 2.6. Pitfalls

Despite its great performance and many advantages, CT angiography is not perfect. It is important to understand the limitations and common pitfalls associated with CT in the evaluation of arterial injuries in order to prevent misinterpretation of the study. Some of the pitfalls include imaging artifacts, bolus-timing issues, and foreign body confusion. With regard to imaging artifacts, volume averaging can lead to the appearance of a change in attenuation in the setting of thicker image reconstructions. In addition, motion artifacts can give the appearance of higher-attenuation contrast in the wrong region of the image. Additional high-attenuation objects, like foreign bodies or bone fragments, can have an appearance similar to some of the injury patterns described above, such as pseudoaneurysm or extravasation. This is especially true if multiple phases of contrast enhancement are not acquired. In addition, these injury patterns are not mutually exclusive and overlapping injury patterns may cause confusion when multiple injuries of the types described above are present. On the contrary, vasospasm may be so intense as to prevent any enhancement of the distal portions of the vessel, which may obscure downstream injuries [5]. In the setting of dissection, thrombosis in the false lumen of a dissection can prevent adequate visualization of a dissection or decrease the apparent extent of injury [16]. In addition, hyperattenuating foreign bodies from the mechanism of injury may result in significant streak artifact [13].

Some of the most important pitfalls involve timing of the contrast bolus. As discussed earlier, a variety of factors can affect the circulation time of contrast-enhanced blood. If flow is especially slow, a rapid scan time can lead to image acquisition moving through the exam faster than the contrast bolus. This results in a portion of the examination being unenhanced. In addition, poor cardiac function can result in suboptimal mixing of contrast, which may prevent the necessary peak attenuation from ever being attained. This results in the image acquisition never being initiated. Likewise, the region of interest may be placed on the incorrect portion of the image, also preventing the triggering of image acquisition. Lastly, bolus tracking cannot be used for smaller arteries, as a large artery is required for a sufficient region of interest to track.

### 2.7. Future Technology

The future of cross-sectional imaging in the setting of arterial injuries is promising. For example, dual-energy CT will have the ability to differentiate iodine from other high-attenuation materials. This would allow for easy differentiation of arterial injuries and could easily visualize poor downstream perfusion following an injury [25]. New techniques for timing of scan acquisition following contrast administration can also prevent some of the non-diagnostic images that may result from some of the described pitfalls. For example, the upcoming technique of bolus chasing may soon be available for CT angiography. Bolus chasing involves tracking the bolus throughout the duration of the scan acquisition and adjusting the table speed based on the propagation of the contrast-enhanced blood. If the bolus begins to travel more slowly, table speed will slow, allowing more time for the bolus to propagate. This is a complicated method of bolus-timing that will likely increase the diagnostic value of CT angiography by reducing the variability inherent to image acquisition following contrast administration [26].

## 3. Conclusions

CT angiography remains the mainstay in cross-sectional imaging of arterial injuries. Despite the known pitfalls, the exam has been shown to have extremely high sensitivity and specificity, regardless of the region of the body being examined. The exam has a variety of tools and techniques that can be utilized for evaluation, including multiple phases of imaging, multiplanar reformatting, maximum intensity projections, and volume rendering. In the coming years, we can look forward to bolus chasing techniques and the benefits of dual-energy CT to build upon our current technology and provide even higher quality imaging and more information than we have available today.
